# Thanatin: An Emerging Host Defense Antimicrobial Peptide with Multiple Modes of Action

**DOI:** 10.3390/ijms22041522

**Published:** 2021-02-03

**Authors:** Rachita Dash, Surajit Bhattacharjya

**Affiliations:** 1 School of Biological Sciences, Nanyang Technological University, Singapore 637551, Singapore; 16immm17@uohyd.ac.in; 2 Department of Systems and Computational Biology, School of Life Sciences, University of Hyderabad, Hyderabad, Telangana 500046, India

**Keywords:** thanatin, multidrug-resistant (MDR) bacteria, antimicrobial peptides (AMPs), lipopolysaccharide (LPS), mechanism of AMPs

## Abstract

Antimicrobial peptides (AMPs) possess great potential for combating drug-resistant bacteria. Thanatin is a pathogen-inducible single-disulfide-bond-containing β-hairpin AMP which was first isolated from the insect *Podisus maculiventris*. The 21-residue-long thanatin displays broad-spectrum activity against both Gram-negative and Gram-positive bacteria as well as against various species of fungi. Remarkably, thanatin was found to be highly potent in inhibiting the growth of bacteria and fungi at considerably low concentrations. Although thanatin was isolated around 25 years ago, only recently has there been a pronounced interest in understanding its mode of action and activity against drug-resistant bacteria. In this review, multiple modes of action of thanatin in killing bacteria and in vivo activity, therapeutic potential are discussed. This promising AMP requires further research for the development of novel molecules for the treatment of infections caused by drug resistant pathogens.

## 1. Introduction

Antimicrobial resistance (AMR) is a growing complex global issue of serious concern. Based on the trends of drug resistance ascent, the O’Neill report [1] estimates that unless appropriate action is taken, AMR will cause up to 10 million deaths annually by the year 2050. In fact, a precarious state of affair is already palpable, as reflected in the research published by Rudd et. al. (2020) [2], which pointed out that 11 million deaths—19.7% of all global deaths in 2017—were related to sepsis [3]. Antibiotics are the front-line treatment for sepsis; however, resistance to many last-resort antibiotics such as carbapenems is frequently occurring in many regions of the world [4]. There is an utmost urgency to establish both therapeutic and preventive solutions to tackle this widespread problem. Yet, progress in the development of antibiotics has been slower than desirable, attributable partially to difficulties when translating laboratory discoveries to the clinic [5,6,7]. Further, in a recent report, the World Health Organization (WHO) highlights the dearth of strong antibiotic candidates and the prevalent weak pipeline of antibiotics—most of which are merely modifications of existing molecules and do not target drug-resistant Gram-negative bacteria [8]. Therefore, a strong emphasis must be laid not only on searching new antibiotics but also on developing innovative alternatives to conventional antibiotics. Novel antibiotics are urgently needed to treat infections caused by the ESKAPE (*Enterococcus faecium*, *Staphylococcus aureus*, *Klebsiella pneumoniae*, *Acinetobacter baumannii*, *Pseudomonas aeruginosa*, *Enterobacter cloacae*) group of pathogens. In 2019, the annual number of antibiotic-resistant infections was estimated to be 2.8 million, resulting in 35,000 deaths in the USA [9]. 

Antimicrobial peptides (AMPs), also known as host defense peptides (HDPs), are a structurally and functionally diverse class of naturally occurring polypeptides that are evolutionarily conserved across all life forms [10,11,12,13,14,15]. AMPs form an integral component of the innate immune system in higher organisms. Apart from being able to target an impressively large spectrum of pathogens encompassing Gram-negative bacteria, Gram-positive bacteria, fungi, and viruses; some of these peptides can also exhibit immunomodulatory effects that indirectly aid in pathogen clearance [16,17,18]. Activities such as anti-infective, anti-inflammatory, wound healing, as well as anti-biofilm properties, can be inherent to some AMPs [19]. Most AMPs are characterized by cationic and hydrophobic residues and employ disruption of the bacterial membrane as a mode of bacterial cell killing, potentially also limiting the development of bacterial resistance [20,21]. Several AMPs and their derived analogs are at different stages of clinical trials, with some even in advanced phases [22]. However, despite the tremendous potential of AMPs to serve as an alternative to conventional antibiotics and relieve to a degree the growing problem of antibiotic resistance, no peptide-based antibiotic has been given regulatory approval so far [19]. The lack of success in clinical translation of this class of molecules can be largely attributed to their poor stability, toxicity to the host, and high production costs [23]. 

## 2. Thanatin

**Figure 1:**
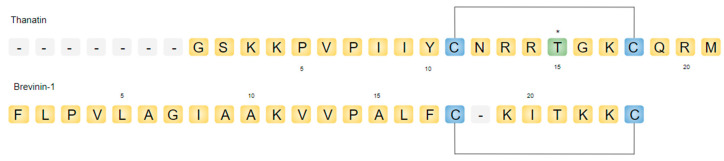
Amino acid sequences of thanatin and brevinin-1. The central Thr residue in the disulfide loop of thanatin is highlighted in green. Cys residues are indicated in blue.

Thanatin is an insect-derived antimicrobial peptide that shows promising effects with respect to its ability to overcome the aforementioned hurdles in the path to clinical success. Thanatin (the name is derived from “thanatos”, i.e., death) is an inducible cationic antimicrobial peptide that was first isolated from the hemolymph of the hemipteran insect *Podisus maculiventris* (spined soldier bug), through immune challenge [24]. The peptide is 21 amino acids long, with the primary structure GSKKPVPIIYCNRRTGKCQRM. Thanatin is strongly cationic (pI of 10.48) and contains a distinct short eight-residue basic loop created through a disulfide bond formation between residues Cys11 and Cys18 at the C-terminus [24] (Figure 1). The S–S loop contains a central threonine amino acid separating two subgroups of positively charged residues [24]. Interestingly, thanatin shares close to 50% overall sequence identity with brevinin-1, a member of the brevinin family of host defense peptides found in frog skin secretions [24]. These peptides have a length of 24 amino acids and are characterized by a seven-residue disulfide ring at their C-terminus [24] (Figure 1). Brevinins have been described to adopt helical conformations in membrane-mimetic environments [25]. On the contrary, thanatin is characterized by a β-hairpin structure in its C-terminal region, retained in both free and detergent solutions, which is considered integral for its activity [26,27]. Remarkably, thanatin is a uniquely multifaceted peptide bestowed with more than one distinct mechanism of antimicrobial activity that is opposed to the commonly observed membrane disruption ability of several cationic AMPs. The existence of such a degree of dimensionality of activity can be desirable in that it may widen the gamut of vulnerable target pathogens. Indeed, thanatin exhibits potent activity against a broad range of pathogens [24]. Fehlbaum et al. (1996) [24] reported activity against Gram-negative bacteria such as *Escherichia coli*, *Salmonella typhimurium*, *Klebsiella pneumoniae*, and *Enterobacter cloacae* with in vitro minimal inhibitory concentrations (MICs) <1.2 µM (Table 1). Interestingly, the d-isomer of thanatin lacks most of its Gram-negative activity, however retaining activity against Gram-positive strains. Weaker activity was reported against *Erwinia carotovora* and *Pseudomonas aeruginosa*. Thanatin was also found to show potent activity against various Gram-positive bacteria such as *Aerococcus viridans*, *Micrococcus luteus*, *Bacillus megaterium*, and *Bacillus subtilis* (MIC < 5µM). Notably, no activity of the native peptide was observed against *Staphylococcus aureus* [24]. Further, thanatin was found to also possess potent antifungal activity (MIC < 5µM) against *Neurospora crassa*, *Botrytis cinerea*, *Nectria haematococca*, *Trichoderma viride*, *Alternaria brassicicola*, and *Fusarium culmorum*. In this review, we discuss the diverse mechanisms of antimicrobial activity of thanatin with special emphasis on structure–activity relationships. We also highlight the in vivo characterization of the peptide and its derivatives, emphasizing all the while the therapeutic potential of thanatin.

## 3. In Vivo Antibacterial Activity of Thanatin

The in vivo efficacy serves as a critical indication of the potential applicability of an antimicrobial peptide. The therapeutic effects of thanatin and many of its derivatives have been extensively studied in vivo with promising outcomes. Thanatin was reported to exhibit efficacy in a mice sepsis model based on infection with New Delhi metallo-beta-lactamase-1 (NDM-1) producing *E. coli* XJ141026, in a concentration-dependent manner. The survival rate of mice increased from 0% in the control group (died within 2 days after infection) to 100% when the mice were treated with 6 mg/kg of thanatin. Thanatin caused a drop in the bacterial titers (collected from tissues 24 h post-infection) and also rescued pathological damages as indicated by histological examination of mice tissues [28]. The bactericidal effects of native and L-thanatin (GSKKPVPIIYCNRRTGKCQRM with free thiols) were analyzed in extended-spectrum β-lactamase-producing *E. coli* (ESBL-EC)-infected mice. The survival rates improved from 16.7% for the control group to 91.7% for the groups treated with 10 mg/kg native thanatin or L-thanatin [29]. C-terminal-amidated thanatin (A-Thanatin, GSKKPVPIIYCNRRTGKCQRM-amidated) is known to have greater tolerance to proteinase when compared to the native thanatin [24]. The in vivo effect of A-thanatin against ESBL-EC has also been ascertained, using a septicemic mice model [30]. On administration of A-thanatin, the survival rate of the mice increased from 0% for the control group to 50.0%, 66.7%, and 91.7% for mice treated with 2.5, 5, and 10 mg/kg of A-thanatin, respectively, along with a decrease in the bacterial titers in mice tissues [30]. In contrast, on treatment with ampicillin, no improvement in the survival rate was seen [30]. S-thanatin (GSKKPVPIIYCNRRSGKCQRM) presents a serine at position 15 instead of the threonine found in the native peptide [31]. The peptide has been shown to exert broad-spectrum antimicrobial activity and is especially potent against Gram-negative bacteria [32]. Assessment of the antimicrobial activity of S-thanatin against a MDR clinical isolate of *K. pneumoniae* (CI120204205) resistant to carbapenems—ertapenem and imipenem—in a septicemic mice model was done. It showed that S-thanatin improved survival rate (from 0% in the control group to 100% in the group treated with 15 mg/kg of peptide) and also lowered the bacterial titers significantly in the intra-abdominal fluid of the animals [33]. Notably, the plasma endotoxin levels were also reduced with S-thanatin treatment [33]. Bacterial biofilms are known to be as much as 1000-fold resistant to conventional antibiotics which are normally used to treat planktonic cells [34]. A shorter derivative of thanatin or R-thanatin (IYNCRRRFCKQRCONH_2_) was designed and examined against methicillin-resistant *Staphylococcus epidermidis* (MRSE) in a urinary tract infection rat model. The peptide caused a decrease of bacterial loads in the bladder and kidney of the experimental animals when administered intraperitoneally. Importantly, biofilm formation on stents implanted in the bladder was also hindered by R-thanatin. Antimicrobial peptides that tend to target the cell membranes of pathogens can also result in hemolytic toxicity which is detrimental to systemic protective effects [35]. Consequently, most of the success obtained in the clinical application of AMPs has remained mainly confined to topical treatments [36,37,38,39,40]. Importantly, thanatin has been shown to be poorly hemolytic even at high concentrations. In particular, both native thanatin and L-thanatin appeared to be non-toxic against human red blood cell (hRBC) suspensions or human umbilical vein endothelial cells (HUVECs) at a concentration 250 times higher than the MIC values [29]. A-thanatin also displayed very low hemolytic activity at concentrations 100 times higher than the MIC values [30]. Further, results from cell toxicity assays performed in human pulmonary alveolar epithelial cells (HPAEpiCs) showed that thanatin had a lower toxicity level than colistin [28]. No toxicity was observed against mouse primary neuron cells as well [28]. These results indicate that thanatin exhibits excellent in vivo efficacy against a broad range of pathogens, with a high degree of selectivity towards bacterial cell membranes over mammalian cell membranes.

### Engineering Thanatin for Superior Activity

The high antimicrobial activity and low toxicity to human cells and tissues of thanatin can be exploited for the development of anti-infective peptides. A comparative study across several disulfide-bonded AMPs—arenicin-3, tachyplesin-1, polyphemucin-1, gomecin, and protegrin-1—revealed that thanatin displayed the lowest RBC lysis activity and cytotoxicity [41]. Surprisingly, the same study also reported higher MIC values of thanatin in antimicrobial assays. Notably, thanatin-derived peptides are being investigated in preclinical studies against systemic fungal infections in immunocompromised patients and MDR bacterial infections [42,43,44]. Despite evidence of favorable activity and toxicity profiles, there insufficient understanding of the structure–activity relationship of thanatin, which limits the design of novel more potent analogs. Primarily, studies have determined the effect of deletions and substitutions of amino acid residues on the activity of thanatin. Analyses of truncated variants of thanatin revealed that N- and C-terminal residues exert different effects on the antimicrobial activity [24]. Table 2 summarizes the antimicrobial activity of thanatin and of its deletion analogs [24]. The sequential removal of the last three residues M21, R20, and Q19 resulted in G20R, G19Q, and G18C analogs. These analogs demonstrated impaired activity in killing Gram-negative bacteria, although retaining much activity against Gram-positive bacteria and several strains of fungi (Table 2). Progressive deletions of residues from the N-terminus of the peptide yielded four analogs—K18M, V16M, I14M, and I12M. The absence of the first three amino acids in the analog K18M did not significantly affect the antibacterial activity of thanatin; however, a slight reduction in its antifungal activity was detected (Table 2). Further deletion of five amino acids in the analog V16M caused greater reduction in the antifungal activity, whereas the antibacterial activity was largely maintained (Table 2). Both Gram-negative antibacterial and antifungal activities were profoundly impaired for the 14-residue I14M analog demonstrating MICs in the 20–40 μM range [24]. The 12-residue-long deletion variant Y12M was devoid of Gram-negative antibacterial and antifungal activities (MICs > 40 μM). The growth of some Gram-positive strains (*A. viridans*, *M. luteus*, *B. megaterium*) was inhibited by Y12M, with MICs in the 20–40 μM range [24]. Collectively, the C-terminal residues of thanatin are more critical for antimicrobial activity than the N-terminal ones. 

The functional roles of residues within the disulfide bond C^11^NRRTGKC^18^ of thanatin were explored by the deletion and insertion of amino acids [45] (Table 3). The antibacterial activity appeared to be somewhat improved against Gram-positive bacteria upon the deletion of residue T15 (Table 3). The deletion of either residue G or residues T and G yielded analogs with rather lowered antibacterial activity (Table 3). These observations pointed out that the shortening of the disulfide loop does not contribute much to the antimicrobial activity of thanatin. Further, an increase in the length of the disulfide loop, by introducing an Ala residue, reduces the antibacterial activity of thanatin (Table 3). The single disulfide bond between residues Cys 11 and Cys 18 in thanatin confers a stable β-hairpin structure in free solution [27,46]. 

Analogs of thanatin lacking the disulfide bond were investigated to understand their relationship with antibacterial activity (Table 4). Disulfide bonds are known to be involved in modulating the activity of β-sheet AMPs [47,48,49]. 

In vitro and in vivo investigation of an analog containing Cys residues in reduced form, as in L-thanatin appeared to retain similar activity profiles akin to native thanatin (Table 4, [29]). However, it can be noted that the experiments with L-thanatin were conducted without the inclusion of any reducing agents. The free thiol groups of the Cys residues of thanatin might inadvertently form an S–S bond under physiological conditions. By contrast, an analog with the two Cys residues replaced by Ala was found to be largely inactive [45] (Table 4). A separate study indicated that chemical modification of the sidechains with the two Cys residues with a tert-butyl group or substitution of Cys with Ser residues impaired the activity against an *E. coli* strain [50]. However, these analogs appeared to retain efficient growth inhibition of Gram-positive *M. luteus* [50] (Table 4). A recombinant overexpression system evaluated *E. coli* cell growth inhibition upon in vivo production of thanatin and several mutants. The C11Y mutation of thanatin resulted in loss of antibacterial activity [51]. S-thanatin, an analog of native thanatin containing a Ser residue in place of Thr15, has been investigated both in vitro and in vivo [31]. Studies demonstrated that S-thanatin displays broad-spectrum antimicrobial activity akin to that of native thanatin [31,32,33]. Thanatin-based hybrid AMPs were also synthesized, and their in vitro antimicrobial activity was reported [52,53,54,55]. Notably, transgenic plants expressing thanatin can become resistant to fungal and bacterial diseases [56,57,58]. 

## 4. Effect of Thanatin on Bacterial Cells, LPS, and Liposome Integrity 

Several studies demonstrated that bacterial cell agglutination could be the mode of action of thanatin. The outer membrane lipopolysaccharide (LPS) of Gram-negative bacteria and the cell wall components of Gram-positive bacteria are the major sites of interactions of thanatin. Recently, bacterial proteins have been identified binding thanatin (vide infra). As observed, the addition of thanatin to cell suspensions of *E. coli* and *M. luteus* resulted in a rapid arrest of cell motility, followed by agglutination into large clumps [24]. Notably, the inner membrane permeability of the bacteria resulted unaltered even at a high concentration (70 μM) of thanatin. However, *E. coli* in the L form, that lack the outer membrane, were found to be less sensitive to thanatin, whereas Gram-positive bacteria in the L form could be killed even at lower doses [59]. Also, the Gram-negative bacterium *S. typhimurium*, expressing a truncated versions of LPS, exhibited greater lethality in the presence of thanatin [43]. Thanatin is able to disrupt the outer membrane of Gram-negative bacteria (*E. coli*) as demonstrated by 1-N-napthylphenylamine (NPN) probe fluorescence [28] and causes outer membrane charge neutralization [27]. Further, the binding of thanatin to the outer membrane can competitively displace the stabilizing Ca^2+^ ions from LPS molecules in the outer membrane [28]. The thermodynamic parameters of LPS–thanatin interactions were estimated from isothermal titration calorimetry (ITC) experiments. Thanatin–LPS complex formation was detected to be enthalpy-driven, indicating the potential involvement of ionic and polar interactions. Thanatin binds to LPS micelles with an estimated K_d_ ranging between 1.5 and 1.09 μM [27,28]. Thanatin induces the aggregation of LPS micelles, and most of its amino acid residues establish close interactions with LPS [27]. The phosphate head groups of LPS, in particular, directly interacts with thanatin. Further, lipid vesicles or large unilamellar vesicles (LUVs) with a composition similar to that of bacterial plasma membranes were also found to undergo agglutination in the presence of thanatin [60]. Interestingly, IR spectra analyses indicated the involvement of Arg residues in the side chains of thanatin in the aggregation process [60].

## 5. Atomic-Resolution Structure of Thanatin in LPS Outer Membrane

**Figure 2:**
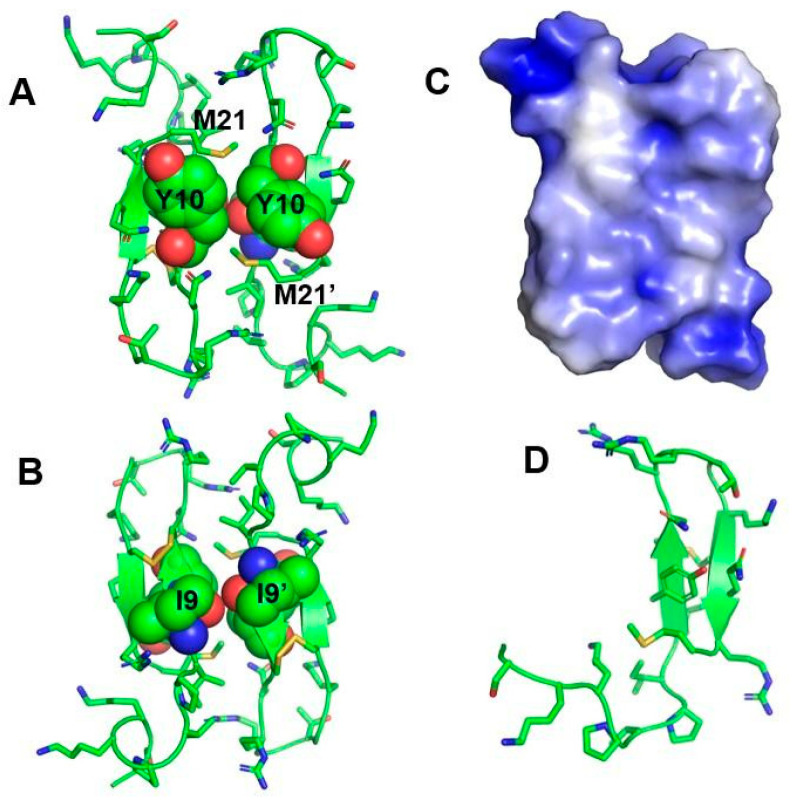
Structures of thanatin in lipopolysaccharide (LPS, pdb:5xo9) and dodecyl phosphocholine (DPC) (pdb: 6aab) micelles. Thanatin forms a dimeric structure in LPS micelles. The side chains of (**A**) Y10/Y10′, M21/M21′, and (**B**) I9/I9′ demonstrated packing interactions in the antiparallel β-sheet topology. (**C**) Electrostatic potential surface of dimeric thanatin. (**D**) Monomeric β-hairpin structure of thanatin in DPC micelles. The β-sheet is shown as a ribbon. The figure was prepared using PyMOL.

Three-D structures of AMPs can be determined in solutions of LPS micelles using tr-NOESY NMR [61,62,63,64]. Also, the LPS-interacting residues of AMPs are determined at atomic resolution by STD-NMR [65,66,67,68]. It is noteworthy that the structures of AMPs in LPS micelles often demonstrate significant differences compared with the structures determined in detergent (sodium dodecyl sulphate or SDS, dodecyl phosphocholine or DPC) micelles [66,69,70]. In addition, 3-D structures and interactions of AMPs in complex with LPS are found to be better correlated with antibacterial activity [71,72,73]. The NMR-derived structure of thanatin in LPS micelles [27] revealed the non-covalent association of two molecules of thanatin in a dimeric organization (Figure 2A). The dimeric topology of thanatin is maintained by four antiparallel β-strands with an interface belonging to the N-terminus of each subunit. Each of the monomeric unit of the dimeric structure assumes a canonical β-hairpin conformation for residues I8–M21, including the disulfide bond, and a mixed conformation (extended and β-turn) for residues G1–I7, at the N-terminal segment. In particular, the residues G1–I7 assumed a definite orientation along with the β-hairpin structure, in that residues K^3^KPV^6^ displayed a β-turn conformation (Figure 2A). The β-turn appears to help in juxtaposing the cationic side chains of residues K3 and K4 with the cationic side chain of residue R20 at the C-terminus of the β-strand (Figure 2A). The anti-parallel four stranded β-sheet structure of dimeric thanatin demonstrates interesting proximity and packing of amino acid residues between the two subunits. Close packing interactions can be realized among side chains of residues Y10/Y10′ and I8/I8′ at the two opposite faces of the β-sheets (Figure 2A,B). Further, the side chains of residues M21 and M21′ are also in close contact with the aromatic sidechain of residues Y10′ and Y10, respectively. Strikingly, the anti-parallel orientation of the dimeric topology of thanatin in complex with LPS micelles permits spatial proximity of distally located cationic residues. Residues R13, R14, and K17, in the β-turn of the β-hairpin, are located on the same side of K3′ and K4′ residues at the N-terminus, yielding large cationic surfaces at the two opposite ends of the structure (Figure 2C). The 3-D structure and orientation of thanatin have recently been reported in zwitterionic DPC micelles (Figure 2D) [26]. In DPC, thanatin assumed a disulfide-stabilized monomeric β-hairpin structure with a flexible N-terminal segment, akin to the structure determined in free solution [26,27]. Several other studies of AMPs also demarcated the striking differences between structures in LPS and in detergent micelles. 

**Figure 3:**
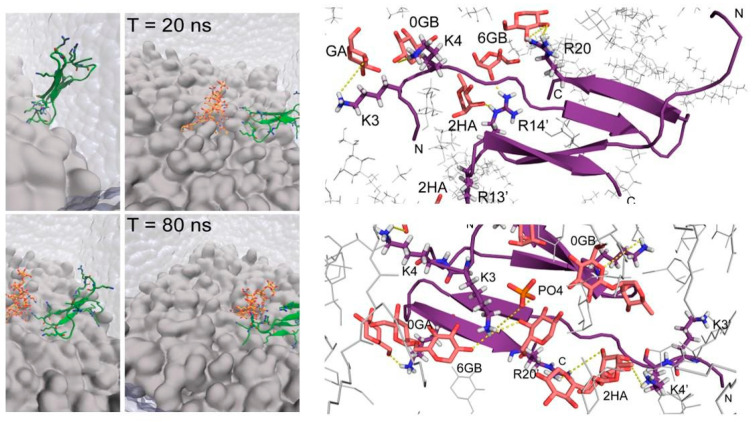
Molecular dynamics (MD) simulation of the interactions between LPS and thanatin. Left panel: time course of the binding of thanatin to LPS in a bilayer observed in the simulation. Right panel: interactions of residues of thanatin with LPS head groups [27].

Molecular modelling and simulations in an LPS/1,2-Dipalmitoyl-sn-glycero-3-phosphoethanolamine lipid (DPEE) bilayer showed that the dimeric thanatin molecule is poised to interact with multiple LPS molecules (Figure 3, left panel, our published data). The 400 ns MD simulation showed a rapid (40–50 ns) binding of thanatin with the LPS layer. The Thanatin/LPS complex formation is largely driven by ionic and/or polar interactions whereby the side chains of the cationic residues K3, K4, and R20 of one subunit and the side chains of the residues R13′ and R14′ of another subunit of the dimer likely engage in interactions with the hydroxyl groups of the sugar residues of LPS (Figure 3, right panel) [27]. In addition, the basic residues K3 and K4 from both subunits of dimeric thanatin appear to establish salt bridge interactions with the phosphate head groups of LPS molecules in the bilayer (Figure 3, right panel). The atomic-resolution structure and mode of interactions of thanatin in complex with LPS can provide valuable mechanistic insights into bacterial cell agglutination. As evident, the dimeric structure of thanatin can efficiently bind to multiple LPS molecules in the outer membrane, which may essentially permit the covering of the bacterial cell surface in a carpet-like fashion. Further, surface charge neutralization and perturbation of LPS outer membrane integrity may perhaps facilitate cell–cell association and agglutination. The dimeric structure of thanatin determined in LPS micelles can be correlated with Gram-negative antibacterial activity. Deletion of the C-terminal residues M21 and R20 of thanatin caused a dramatic reduction of its activity [24]. Ala scanning mutational analyses of the residues Y10, M21, R13, and R14 revealed functional importance [27]. As seen, these residues are either involved in direct interactions with LPS or critical for the dimeric folding of thanatin.

## 6. Binding of Thanatin with LPS Transport Protein Complex and Interactions with Metallo-β-Lactamase of Gram-Negative Bacteria

**Figure 4:**
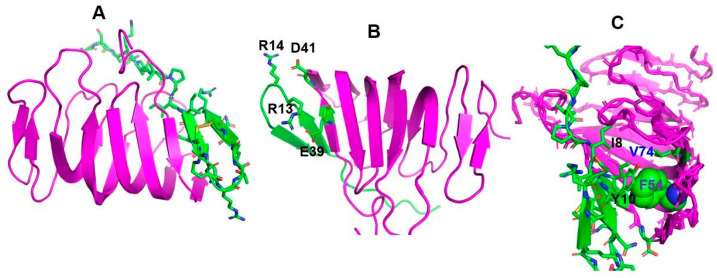
Structure (pdb: 6GD5) of thanatin in complex with LptA_m_: (**A**) The 3-D topology of the complex showing the β-sheet jelly-roll fold (purple) of LptA_m_ and the β-hairpin of thanatin (in green). The side chains of residues of thanatin are shown as sticks. (**B**) Potential ionic interactions of residues R13 and R14 of thanatin with residues E39 and D41 of LptA_m_. (**C**) Packing interactions of residues Y10, I8 of thanatin with hydrophobic-pocket residues F54 and V70 of LptA_m_.

A recent study demonstrated the binding of thanatin with periplasmic proteins involved in LPS transport to the outer membrane. This has been postulated to be the principal mode of action of thanatin in bacterial killing [74]. A large protein complex termed LptA–G, comprised of seven components, is known to be involved in transporting LPS from the bacterial inner membrane to the surface outer membrane. Photoaffinity labeling of thanatin with bacterial proteins identified three proteins, namely, LptA, LptD, and BAM B, of which LptA and LptD were deduced to be abundantly present. The binding of thanatin with the two Lpt proteins was estimated to be of high affinity, with K_d_ values ranging from 12 to 20 nM and 34 to 44 nM for LptA and LptD, respectively. The smaller size of LptA_m_ (a truncated version of LptA) made it amenable for NMR structure determination in complex with thanatin (Figure 4). The LptA_m_–thanatin complex revealed that the N-terminal region of thanatin forms an interacting interface with LptA_m_. In particular, the N-terminal β-strand (residues P7–N12) of thanatin docked onto the first N-terminal β-strand (residues P35–S40) of LptA_m_ (Figure 4). The side chains of the residues Y10 and I8 of thanatin can be seen buried inside a hydrophobic pocket, consisting of the side chains of residues Ile38, Leu45, Val52, Phe54, Val74, and Ile86 of the β-sheet jelly-roll structure of LptA_m_ (Figure 4). Also, the side chains of the non-polar residues V6 and P7 of the N-terminal tail of thanatin make additional hydrophobic packing interactions with the residues P35 and I36 of LptA_m_ (Figure 4). These interactions have been thought to stabilize the thanatin/LptA_m_ complex. The C-terminus β-strand and β-turn residues of thanatin appeared not to interact with LptA_m_ and were solvent-exposed. A recent study indicated that thanatin is able to better inhibit LptA and LptC interactions compared to the homotypic complex LptA/LptA [75]. Further, *E. coli* cells treated with sub-lethal concentrations of thanatin demonstrated degradation of LptA, followed by the accumulation of colanic acid in the outer membrane. However, a mechanistic understanding of thanatin/LptA interactions is yet to be reported. Interestingly, isolated mutations (Q62L, D31N) in LptA conferring resistance to thanatin have been mapped outside the thanatin/LptA_m_ interface. Rather, Ala mutational analyses of thanatin could be correlated with LPS binding and Gram-negative antibacterial activity. Our studies demonstrated that the replacement of residues R13 and R14 with Ala (thanatin R13R14AA) caused loss of antibacterial activity [27]. The R13R14AA thanatin analog was inefficient in surface charge neutralization and lacked interactions with LPS outer membrane. Another analog of thanatin, Y10M12AA, exhibited lower antibacterial activity and limited surface charge neutralization of *E. coli* cells. Regardless, it is noteworthy that residues belonging to the N-terminal β-strand of the β-hairpin and the N-tail region of thanatin were also present at the interface in the dimeric structure of thanatin in LPS micelles (Figure 2). It is likely that the N-terminal region of thanatin may in general behave as a protein–protein interaction surface, which can be exploited for engineering novel functionalities. Further, thanatin efficiently killed antibiotic-resistant *E. coli* strains producing the New Delhi metallo-β-lactamase-1 or NDM-1, both in vivo and in vitro [28]. Remarkably, thanatin demonstrated outer membrane permeabilization, LPS binding, and direct interactions with the enzyme NDM-1, with an estimated K_d_ of 0.71 μM. It has been postulated that thanatin binds near the active site of NDM-1, replacing divalent Zn^+2^ ions required for enzymatic function. However, atomic resolution data for the thanatin/NDM-1 complex are yet to be reported. 

## 7. Mode of Gram-Negative Bacterial Cell Killing by Thanatin 

Based on recent studies, as discussed above, the LPS outer membrane and LPS translocation protein complexes are important targets of thanatin in Gram-negative bacterial cell killing. The dimeric structure of thanatin deduced in LPS micelles essentially suggests the molecular mechanism of outer membrane disruption [27]. The dimeric state of thanatin with augmented cationicity and amphipathicity would be pivotal for outer membrane permeabilization and efficient surface charge neutralization. Ala substitutions of critical residues of thanatin are well correlated with reduced affinity to LPS and concomitant lack of bacterial cell killing [27]. Permeabilized and charge-neutralized LPS outer membrane, bound with dimeric thanatin, can efficiently induce the cell agglutination process, eventually leading to bacterial cell death. Once disrupted, the outer membrane would allow thanatin, presumably in monomeric form, to gain access into the periplasmic space, enabling its interaction with Lpt protein complexes and causing the inhibition of LPS translocation to the outer membrane. The lack of transport of LPS to the outer membrane perhaps would further affect outer membrane stability and permeability, contributing to the cell agglutination process. 

## 8. Conclusions

The broad-spectrum antimicrobial activity of thanatin in conjunction with its low cytotoxicity and high in vivo stability need to be exploited for treatments against infection caused by MDR pathogens. However, there is a significant dearth of research focused on the development of thanatin-based potent AMPs. In particular, the structure–activity relationship of thanatin has yet to be well investigated. It remains unclear how sequence and structural modifications of thanatin can be translated into highly active AMPs. Further, the mode of action of thanatin is ambiguous at present. The exact mechanisms of bacterial cell killing, membrane permeabilization, and intracellular proteins targets require future investigation through mutational analyses and structure determination of thanatin in complex with bacterial targets. The lethality mechanisms of thanatin for Gram-positive bacteria and fungi are largely elusive, since these microorganisms do not contain LPS or LPS translocation protein complexes. In particular, thanatin is an attractive host defense peptide that demands significant incisive work in the fight against MDR pathogens. 

## Figures and Tables

**Table 1:** Antimicrobial activity of thanatin against bacteria and fungi [24].

Microorganism	MIC (µM)
**Gram-Positive Bacteria**	
*Aerococcus viridans*	0.6–1.2
*Micrococcus luteus*	1.2–2.5
*Bacillus megaterium*	2.5–5
*Bacillus subtilis*	2.5–5
*Staphylococcus aureus*	No activity
*Pediococcus acidolactici*	20–40
**Gram-Negative Bacteria**	
*Escherichia coli* D22	0.3–0.6
*E. coli* D31	0.3–0.6
*E. coli* 1106	0.6–1.2
*Salmonella typhimurium*	0.6–1.2
*Klebsiella pneumoniae*	0.6–1.2
*Enterobacter cloacae*	1.2–2.5
*Erwinia carotovora*	10–20
*Pseudomonas aeruginosa*	20–40
**Fungi**	
*Neurospora crassa*	0.6–1.2
*Botrytis cinerea*	1.2–2.5
*Nectria haematococca*	1.2–2.5
*Trichoderma viride*	1.2–2.5
*Alternaria brassicola*	2.5–5
*Fusarium culmorum*	2.5–5
*Ascochyta pisi*	5–10
*Fusarium oxysporum*	10–20

MIC: minimal inhibitory concentration.

**Table 2:** Primary structures and antimicrobial activity of truncated variants of thanatin. G: Gram

Sequence	(G−) Activity	(G+) Activity	Antifungal
GSKKPVPIIYCNRRTGKCQRM (Thanatin)	++++	++++	++++
GSKKPVPIIYCNRRTGKCQR (G20R)	+	+++	+++
GSKKPVPIIYCNRRTGKCQ (G19Q)	−	++	+++
GSKKPVPIIYCNRRTGKC (G18C)	−	++	+++
KPVPIIYCNRRTGKCQRM (K18M)	++++	++++	+++
VPIIYCNRRTGKCQRM (V16M)	+++	+++	++
IIYCNRRTGKCQRM (I14M)	+	++	+
YCNRRTGKCQRM (Y12M)	−	+	−

**Table 3:** Primary structures and antimicrobial activity of deletion and insertion variants of thanatin.

Sequence	(G−) Activity	(G+) Activity
GSKKPVPIIYCNRRTGKCQRM (Thanatin)	++++	++++
GSKKPVPIIYCNRR-GKCQRM (Del T)	++++	+++++
GSKKPVPIIYCNRRT-KCQRM (Del G)	+++	+++
GSKKPVPIIYCNRR-KCQRM (Del T, G)	+++	+++
GSKKPVPIIYCNRRATGKCQRM (Ins A)	++	++
GSKKPVPIIYCNRRAATGKCQRM (Ins AA)	++	++

**Table 4:** Role of the disulfide bond in the antimicrobial activity of thanatin.

Sequence	(G−) Activity	(G+) Activity
GSKKPVPIIYCNRRTGKCQRM (Thanatin)	+++	+++
GSKKPVPIIYCNRRTGKCQRM (L-Thanatin)	+++	+++
GSKKPVPIIYANRRTGKAQRM (C to A)	−	−
GSKKPVPIIYSNRRTGKSQRM (C to S)	−	+++
GSKKPVPIIYXNRRTGKXQRM (C to X, X stands for Cys residues modified with tert-butyl group)	−	+++
